# Lessons and recommendations from three decades as an NSF REU site: A call for systems‐based assessment

**DOI:** 10.1002/ece3.6136

**Published:** 2020-03-02

**Authors:** Andrew L. McDevitt, Manisha V. Patel, Aaron M. Ellison

**Affiliations:** ^1^ Harvard Forest Harvard University Petersham MA USA; ^2^ Department of Integrative Biology University of Colorado Denver Denver CO USA; ^3^ Sound Solutions for Sustainable Science Boston MA USA

**Keywords:** cultural–historical activity theory, Harvard Forest, research experiences for undergraduates, STEM, undergraduate research experience

## Abstract

For more than 30 years, the US National Science Foundation's Research Experiences for Undergraduates (REU) program has supported thousands of undergraduate researchers annually and provides many students with their first research experiences in field ecology or evolution. REUs embed students in scientific communities where they apprentice with experienced researchers, build networks with their peers, and help students understand research cultures and how to work within them. REUs are thought to provide formative experiences for developing researchers that differ from experiences in a college classrooms, laboratories, or field trips. REU assessments have improved through time but they are largely ungrounded in educational theory. Thus, evaluation of long‐term impacts of REUs remains limited and best practices for using REUs to enhance student learning are repeatedly re‐invented. We describe how one sociocultural learning framework, cultural–historical activity theory (CHAT), could be used to guide data collection to characterize the effects of REU programs on participant's learning in an educationally meaningful context. CHAT embodies a systems approach to assessment that accounts for social and cultural factors that influence learning. We illustrate how CHAT has guided assessment of the Harvard Forest Summer Research Program in Ecology (HF‐SRPE), one of the longest‐running REU sites in the United States. Characterizing HF‐SRPE using CHAT helped formalize thoughts and language for the program evaluation, reflect on potential barriers to success, identify assessment priorities, and revealed important oversights in data collection.

## Graphical abstract

This article proposes using cultural–historical activity theory (CHAT) as a systems‐based theoretical framework for evaluating and improving experiential undergraduate research programs. After describing the CHAT framework, we illustrate its application to the collection and interpretation of data collected by the Harvard Forest Summer Research Program on recruitment and student hiring practices; understanding variation in learning gains; and assessing its long‐term impacts.

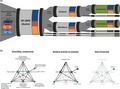

## 1 INTRODUCTION

**Figure 1:**
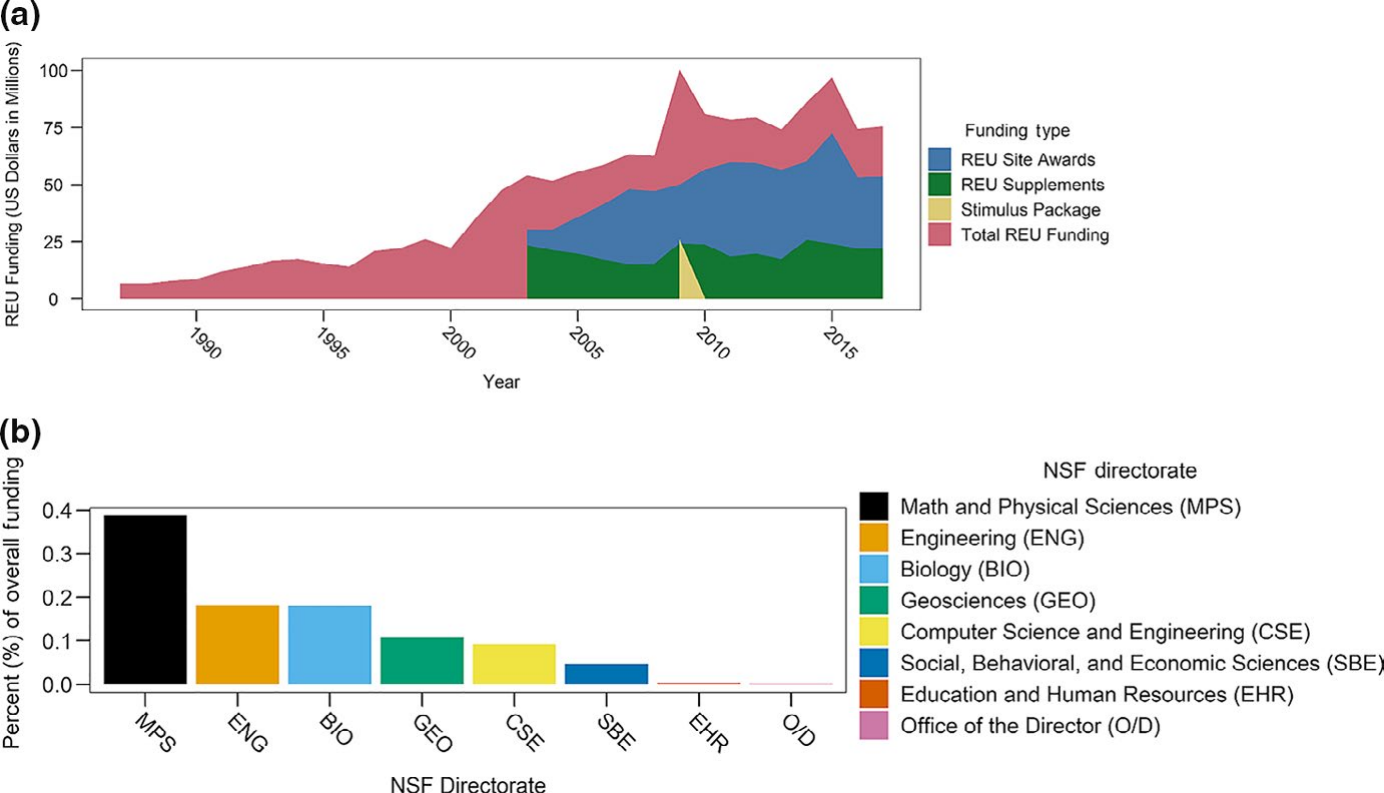
Funding for Research Experiences for Undergraduates (REU) programs. Support for REU programs based on (a) yearly congressional allocations and (b) NSF directorate support for REU sites. Funding data (2002–2017) were compiled based on yearly NSF congressional budget requests. Archives of REU awards (nsf.gov/awardsearch/) provided estimates for remaining years and directorate contributions

Undergraduate research experiences in research laboratories and at field stations or remote field sites strengthen student preparation within scientific disciplines (Kuh, 2008). One of the first programs to support such experiences in the United States was the Undergraduate Research Participation Program (1958–1982), through which the US National Science Foundation (NSF) supported paid student internships across the sciences (Neckers, 1982). In 1987, NSF resumed supporting undergraduate research through the Research Experiences for Undergraduates (REU) program. Since then, REU has become one of the largest supporters of undergraduate research programs; $1.12 billion was invested in supporting thousands of undergraduates each year between 2002 and 2017 through both REU Site and REU Supplement awards (Figure 1).

All REU Site and REU Supplement awardees share the common goal of preparing undergraduate students for careers in science, technology, engineering, and mathematics (STEM) fields by providing research opportunities. Our focus here is on REU sites, which support cohorts of six or more students working with more than one senior researcher and that explicitly include educational programming beyond the field or laboratory research itself. Individual REU sites are defined uniquely by their intellectual themes (approximately 10% related to ecology or evolution) and communities of researchers. The design of educational experiences at each REU site depends on these themes and the values articulated by program leadership and individual scientists. Sites vary in their personnel, infrastructure, intellectual pursuits, and the student populations they serve. Sites also vary in how they evaluate their goals and assess their success.

At least through 2010, if individual REU sites evaluated and assessed themselves at all, they selected and managed their own assessment protocols. Individual site assessments generally were unique case studies (Dávila, Cesani, & Medina‐Borja, 2013) derived from internally developed participant surveys (McDevitt, Patel, Rose, & Ellison, 2016; Seymour, Hunter, Laursen, & DeAntoni, 2004) administered only after the program ended. Qualitative data from these surveys elicited insights about student experiences but the data were neither representative nor a random sample and were expensive to collect. More widely used quantitative surveys created less of a burden on programs, but the surveys often consisted of conceptually ambiguous questions that rarely were validated and were incomparable among programs (Linn, Palmer, Baranger, Gerard, & Stone, 2015; McDevitt et al., 2016).

NSF has supported development of assessment tools to evaluate the effectiveness of REU sites in meeting national‐level goals. By the early 2000s, data collected by NSF revealed that undergraduate research programs successfully recruited women (Kardash, 2000; Liang, Tracy, Taylor, & Williams, 2002) and minority students into STEM fields (Foertsch, Alexander, & Penberthy, 2000; Gregerman, Lerner, Hippel, Jonides, & Nagda, 1998; Nnadozie, Ishiyama, & Chon, 2001). In 2003, NSF aligned REU program goals with these findings and prioritized REU sites over supplements in their annual budget (Figure 1a). The passage of the America COMPETES Act of 2010 (US P.L. 111‐478 §514) further strengthened initiatives to reach diverse participants, especially from institutions where STEM research opportunities are perceived to be limited. It also mandated the tracking of matriculation into STEM fields by REU participants and their subsequent employment for at least 3 years following graduation from community (2‐year) colleges, undergraduate (4‐year) colleges, or universities. At the same time, REU sites focused on research in the biological sciences (BIO) began using a common assessment tool, the Undergraduate Research Student Self‐Assessment (URSSA; Hunter, Weston, Laursen, & Thiry, 2009), to evaluate common goals and improve communication about BIO‐REU programs (Christian & Hannigan, 2010).

The flexibility afforded to REU sites by NSF encourages innovative pedagogical approaches but also increases heterogeneity among programs. In contrast, surveys such as URSSA were developed to assess programmatic goals prioritized by NSF. Both individual site‐based surveys and cross‐site surveys like URSSA serve their intended purpose, but both lack theoretical underpinnings that make it difficult to relate their findings to the broader literature on education or to understand similarities and differences among REU sites (Beninson, Koski, Villa, Faram, & O'Connor, 2011; Linn et al., 2015; Wilson et al., 2018).

Our own experience suggests that using atheoretic assessment tools makes it difficult to understand why an REU program is successful. We previously analyzed 10 years of before/after (“pre/post”) surveys of student participants in the Harvard Forest Summer Research Program in Ecology, which has been supported continuously by NSF as an REU site since 1989 (McDevitt et al., 2016). The design of our short self‐reporting survey was an intentional compromise between sample size and survey depth, and we asked questions about topics we as scientists thought were important rather than those that educators might have identified as central to learning science. The former included changes in students’ attitudes toward science; identification with scientific norms and professional practices; specific skills associated with conducting and disseminating scientific research; and postprogram career and educational plans. We observed significant differences in learning gains correlated with students’ prior experiences in classrooms, laboratories, or the field, but we were unable to attribute causes to these observations or compare our results with similar observations at other sites (e.g., Scott et al., 2012).

These experiences led us to consider aligning our assessment tools with established educational frameworks and theories. Here, we present one such systems‐based framework—cultural–historical activity theory (CHAT)—which we think would be useful for assessing and evaluating REU sites both singly and together. We illustrate how we have begun to apply the CHAT framework to study and improve our own REU site at the Harvard Forest. We suggest that by framing questions as testable hypotheses, results of REU evaluations and assessments can be used to adaptively improve individual undergraduate research experiences and illuminate causes of successes—and failures—across REU sites in ecology, evolution, and other STEM fields.

## 2 USING A SYSTEMS‐BASED APPROACH TO STUDY REU SITES

Ecologists have long recognized the complexity of biological systems and have developed techniques and models—“systems thinking”—to study the interconnected components that make up these systems (Patten & Fath, 2018; Patten & Odum, 1981; Trewavas, 2006). Key features of ecological systems include hierarchical structure, interconnectedness between system components, and emergent properties. REU programs are similarly complex, and by extension, we suggest that systems thinking could be applied to understand and evaluate REU programs if relevant system components could be identified and adequately contextualized.

At REU sites, groups of students engage in research guided by an experienced researcher or laboratory group. REU goals usually extend beyond learning research skills and completing a research project. They also aim to promote the development of scientific identity and cultural capital. Students not only are mentored in research, but they also are connected to a community of peers who can help them navigate through their research and life experiences. In such collaborative learning experiences, paths to success differ among students, cohorts, and programs. Context is very important for understanding both why a program is successful and how to transfer successful practices across programs.

Many learning theories recognize social and cultural influences on learning. A common property among most sociocultural learning theories is that learning is culturally mediated: words, texts, social cues, and other symbolic objects fundamentally shape how an individual constructs knowledge (e.g., Vygotsky, 1980; Wertsch, 1993). Although there is considerable overlap among sociocultural learning theories, most include at least one of three themes. First, learning is less about accumulation of knowledge than performance in different social contexts (“situated cognition”: Brown, Collins, & Duguid, 1989; “embodied cognition”: Wilson, 2002). Second, knowledge is co‐constructed with other individuals or psychological tools (“situated learning”: Lave & Wenger, 1991; “distributed intelligence”: Pea, 1993; “socially shared cognition”: Resnick et al., 1991; “distributed cognition”: Salomon, 1997). And third, the environment, community, or culture shapes how an individual learns (“bioecological theory of human development”: Bronfenbrenner & Morris, 2006; “cultural psycology”: Cole, 1998; “activity theory”: Engeström, Miettinen, Punamäki, & eds., 1999; “cultural learning”: Tomasello, Kruger, & Ratner, 1993; “cultural–historical activity theory”: Roth & Lee, 2007). Each of these sociocultural learning theories provides a slightly different perspective on learning and the context of a research question determines the selection of a theoretical framework (or competing frameworks). Among these, cultural–historical activity theory (CHAT; Roth & Lee, 2007) includes all three themes and flexibly accommodates most concepts proposed in the other sociocultural learning theories. Thus, we consider it to be an ideal platform for a well‐structured assessment of REU programs.

## 3 CULTURAL–HISTORICAL ACTIVITY THEORY

CHAT provides a broad blueprint describing the components that influence the social construction of knowledge (Cole & Engeström, 1993). It is an expansion of activity theory that allows researchers to study the completion of goals by individuals or collaborative groups while recognizing interacting cultural and historical influences acting on the system (Roth & Lee, 2007). Activity theory as a framework for learning builds from a core tenet of cultural psychology (Cole, 1998): The process of learning by an individual can be culturally mediated (Wertsch, 1993). Activity theory is distinguished from other sociocultural learning theories through its explicit identification of the tools an individual uses to learn, how other individuals mediate learning through cultural norms, and the examination of their interactions (i.e., an activity system). The cultural–historical aspect of CHAT extends analysis of an activity system to understand how the activity develops and changes over time and how it relates to other activity systems with which an individual interacts.

### 3.1 Visualizing CHAT systems

**Figure 2:**
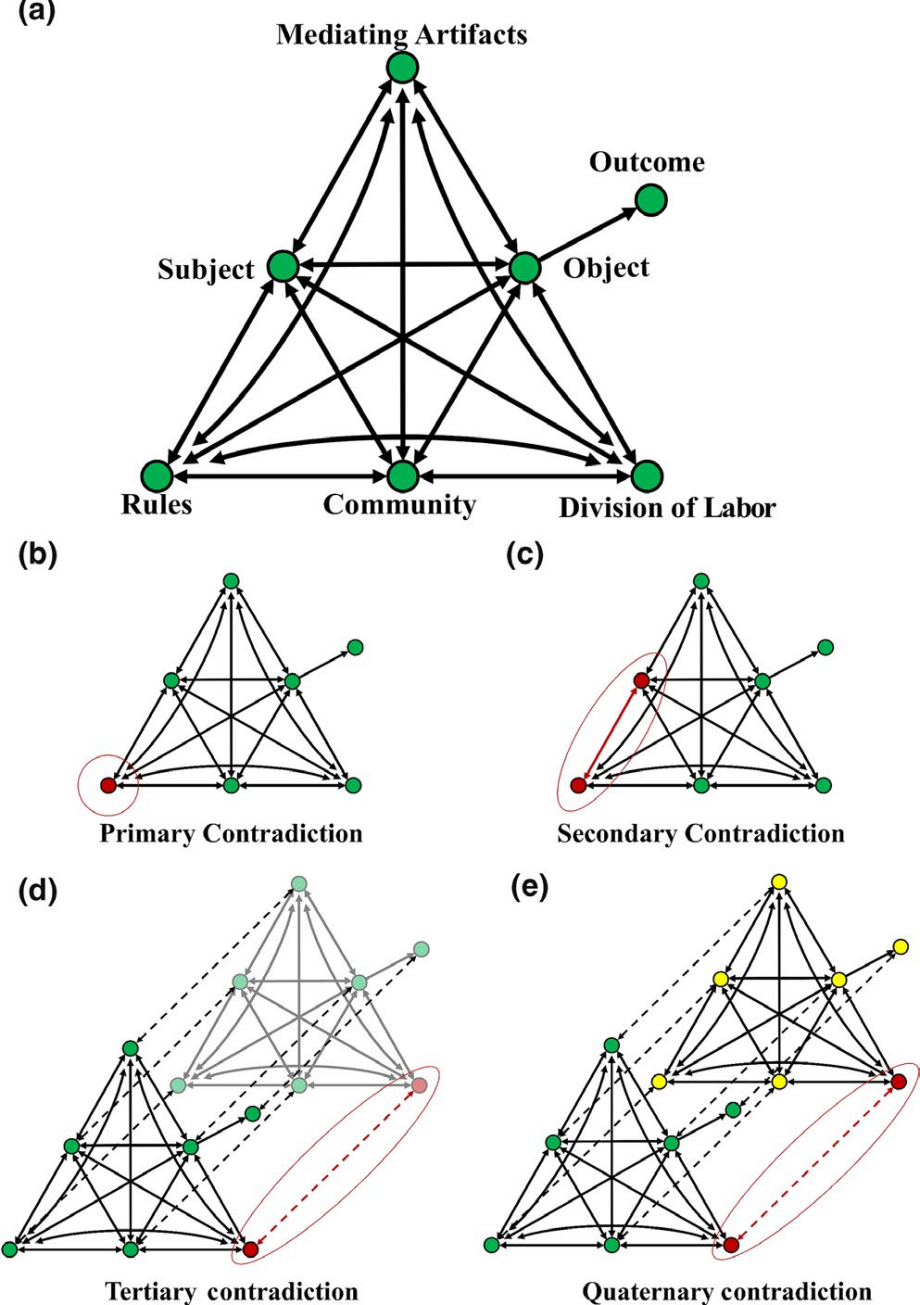
System components of the cultural–historical activity framework (CHAT). The activity triangle highlights how components interact with others within the system (top), and the contradictions that can be examined through CHAT

Cultural–historical activity theory's activity systems are best visualized through what are known as “activity triangles” (Figure 2; Roth & Lee, 2007). CHAT requires the identification of seven distinct elements (“nodes”) that take part in an activity within a system of interest and the examination of connections (“edges”) between them (Cole & Engeström, 1993; Roth & Lee, 2007; Yamagata‐Lynch, 2010). To help our colleagues cut through the educational jargon associated with CHAT, we illustrate its elements in the context of describing a student writing a research proposal:

1. *Subject—*The individual or group of focus during the specified activity (e.g., the undergraduate student(s) writing the proposal);
2. *Object—*The goal or motive behind the specified activity (e.g., students should think critically about their project, connect with the primary literature, and establish feasible milestones for it);
3. *Rules—*The stated or unstated rules that govern how individuals act within the context of the specified activity (e.g., proposal guidelines, conventions of scientific writing, laboratory expectations, or culture as established by research mentor);
4. *Community—*The social context in which the specified activity is conducted (e.g., including the student, research mentor, members of a laboratory, broader group of student participants);
5. *Division of labor—*How tasks are shared among the community to accomplish the specified activity (e.g., the student is responsible for most of the writing, the mentor provides some direction and feedback, and other laboratory members are available to answer questions);
6. *Mediating artifacts—*The tools used in creating or completing the *object* (e.g., example project proposals, relevant journal articles, attending workshops, written feedback);
7. *Outcome—*The effect generated by subject working in concordance with other components of the activity system to accomplish the *object* (e.g., formal evaluation of written proposal, performance review based on expectations outlined in proposal, gaining a skill).

### 3.2 Using CHAT to make sense of contradictory information in complex learning environments

REU programs are complex social learning environments, and CHAT provides the ability to make sense of contradictory information that arises within the system and through time (Cole & Engeström, 1993). These contradictions are classified into four types (Engeström, 1987): *primary contradictions* exist within an element (e.g., contradictory *rules*); *secondary contradictions* exist within interactions between two elements (e.g., *division of labor* is not aligned with *mediating artifacts*); *tertiary contradictions* are manifested during temporal transitions of an activity system (e.g., mentors refining or modifying their approach “on the fly” while the student is writing their research proposal); and *quaternary contradictions* exist between similar activity systems of which the subject is a member (e.g., REU experience compared to scientific coursework).

Primary contradictions often result from differing value judgments that underlie the system (Engeström, 1987). These contradictions are fundamental to the system and form the foundation of higher orders of contradictions (Engeström, 1987; Foot & Groleau, 2011). After program values are established, components within an activity system should be aligned to aid the *subject* in accomplishing the *object*, measured by the *outcome(s)*. For example, in developing a research proposal, the student (*subject*) should be supported in a way that helps them write a successful research proposal (*object*) that is measured by the expectations set by their research mentor or review panel (*outcome*)*.* However, it is common that two or more of these components are not aligned.

Secondary contradictions help to illuminate these misalignments and may lead to subsequent changes within the activity system (Engeström, 1987). For example, an undergraduate student (*subject*) writing a research proposal (*outcome*) may not possess the necessary background knowledge to read a highly technical literature review on their topic (*mediating artifact*); the research mentor or other laboratory members (*community*) may not have enough time to adequately support the student by answering questions and providing feedback (*division of labor*); or expectations conveyed via a micromanagement approach (*rules*) conflict with the ability for the student to meaningfully connect with the literature or think independently about their project (*object*). These conflicts between system components may result in specific obstacles that are manifestations of fundamental tensions (*primary contradictions*) within the activity system (Foot & Groleau, 2011). Because conflicts and contradictions may arise from fundamental components of the system, it is better to address their source(s) rather than their symptoms. To resolve *secondary contradictions* by addressing underlying *primary contradictions*, some type of change must occur in the activity system. For example, before trying to develop new *mediating artifacts* to help a student read a highly technical literature review (*secondary contradiction*), it would be prudent to first evaluate whether there already are m*ediating artifacts* in place that send conflicting messages (*primary contradiction*), which once addressed, might resolve the *secondary contradiction*.

*Tertiary contradictions* are differences in the system that occur at temporal transitions (Engeström, 1987); program directors may be interested in examining them as they change various instructional activities or procedures. For example, an REU program may implement a new proposal‐writing workshop (*mediating artifact)* that is intended to help students (*subject)* connect their proposals to the available scientific literature (*outcome*) and simultaneously shift some of the duties from the research mentor to the workshop facilitator and the student's peers (*division of labor*)*.* As new procedures are implemented, a transition to more “advanced” practices may not be immediate (Engeström, 1987; Foot & Groleau, 2011). Examining barriers to change may reveal additional information about *primary contradiction*s and potentially lead to smoother tertiary transitions.

Alternatively, the cause of these underlying contradictions may not reside solely within the activity system itself, but rather may be rooted in cultural expectations from adjacent activity systems (*quaternary contradictions*). Students (*subjects*) bring their past experiences with them to the activity system, and it is likely that members of the *community* may not have the same shared experiences. For example, the *rules* established in adjacent activity systems may carry over for an individual and impact how said individual interacts with system components such as *mediating artifacts* or the *community.* For example, if a student (*subject*) has prior experience writing a research proposal (*object*) in another context (e.g., in a different laboratory, discipline, or institution), their perceptions of this current experience in writing may be influenced by *rules, mediating artifacts*, or *division of labor* from their other experience (*adjacent activity system*). In this case, the success in writing their REU research proposal (*outcome)* is driven by the recognition of these *quaternary contradictions* and relevant interventions, such as the adjustment of *rules*, addition of *mediating artifacts*, or changes to the *division of labor* that can lead to more productive writing process by the student (*subject*).

## 4 APPLYING THE CHAT FRAMEWORK TO THE REU EXPERIENCE

To help REU programs connect program evaluations with the CHAT framework, we have developed some guiding questions related to activity system components and contradictions (Table 1). These questions are intended to elicit values and perspectives that might not be included in atheoretic surveys or other assessment tools. After fully characterizing the activity system of interest, we prioritized data collection efforts based on our understanding of program values, the magnitude of impact contradictions could have on the activity system, and plausibility of contradictions occurring. We then suggest a rubric (Table 2) to evaluate the strength of evidence for each activity system component and contradiction. Through three examples of applying CHAT to REU assessment (Tables 3, 4, 5), we specifically reflect on data we have collected in the last five years aimed at examining the alignment between our program priorities and current assessment practices.

**Table 1:** Guiding questions for characterizing CHAT system components

System component	Identifying components	Identifying contradictions
Subject	*S.0a* – As the evaluator, how would you characterize the subject(s)? *S.0a* – How might the subjects characterize themselves?	*S.1* – Is there a consensus about the characterization of the subject? What type of information might be relevant to the activity system, and how might you go about collecting this description?
		*S.2* – See below. Secondary contradictions will be examined once other components of the activity system have been examined.
		*S.3* – If you are studying this activity system over time, how has the characterization of your subject changed over time?
		*S.4* – What are some similar activity systems the subject may be involved in?
Object	*Ob.0a* – What is the activity/practice you want to evaluate? *Ob.0b* – How would the subject characterize the object? *Ob.0c* – Are there other members of the community that would characterize the object differently?	*Ob.1* – Is there a clear consensus about the goals of the activity/practice under evaluation? If not, where might you expect to find additional contradictions?
		*Ob.2* – How does the subject perceive the object? (e.g., Do they have the same priorities?)
		*Ob.3* – If you are studying this activity system over time, how has the object changed over time?
		*Ob.4* – What are additional activity systems might have similar objects/goals?
Outcome	*Out.0a* – What values or perspectives are important to recognize when assessing the object? (e.g., student “success” may mean many things to different people) *Out.0b* – How do you plan on assessing the object?	*Out.1* – Why is this outcome measurement appropriate for the object? What are the documented validity and reliability arguments for this object?
		*Out.2* – What evidence do you have that the outcome measures are aligned with your object?
		*Out.3* – If you are studying this activity system over time, how have your outcome measures changed? (e.g., refinement or development of instruments/surveys)
		*Out.4* – If you are using an instrument that was developed to under a different context/population, how might this impact the validity and reliability arguments?
Community	*C.0* – Who interacts with the subject to accomplish the object?	*C.1* – Does the subject acknowledge or recognize the rest of the members of the community?
		*C.2* – Do both the subject and community know who is involved in the activity system?
		*C.3* – If you are studying this activity system over time, how has the community changed?
		*C.4* – How does this community compare to similar activity systems? What is the impact these differences have on the outcome?
Rules	*R.0* – What rules, expectations, or cultural norms are present that potentially impact how the subject interacts with the activity system?	*R.1* – Are any rules or norms at odds with one another? (i.e., can they send conflicting messages)
		*R.2a* – Are any rules in direct conflict with the object?
		*R.2b* – Do subjects or community members all value or agree with the rules of the system?
		*R.3* – If you are studying this activity system over time, how have rules changed over time?
		*R.4* – How do the rules of this activity system differ from similar activity systems? What impact do these differences have on the outcome?
Mediating artifacts	*MA.0* – What tools or practices are used help the subject achieve/accomplish the object?	*MA.1* – What evidence can be provided there that the various mediating artifacts are aligned?
		*MA.2a* – How does the use of mediating artifacts align with the object?
		*MA.2b* – How does the subject perceive the utility of individual mediating artifacts?
		*MA.2c* – How does the community perceive the utility of individual mediating artifacts?
		*MA.2d* – Are any rules in conflict with mediating artifacts (or with how the community divides the labor with these artifacts)?
		*MA.3* – How did the addition, removal, or refinement of mediating artifacts impact the activity system?
		*MA.4* – How do these mediating artifacts compare to similar activity systems? What is the impact these differences have on the outcome?
Division of labor	*DL.0* – What are the expectations of the community to be involved in helping achieve the object?	*DL.1* – What evidence can be provided to demonstrate that community members are achieving these expectations?
		*DL.2a* – Is the division of labor appropriate to meaningfully support individual mediating artifacts?
		*DL.2b* – Is the overall division of labor in the activity system appropriate to meaningfully support the object?
		*DL.3* – If you are studying this activity system over time, how has the division of labor changed over time?
		*DL.4* – How does the division of labor differ from similar activity systems? What is the impact these differences have on the outcome?

**Table 2:** Recommendations for rating the quality of evidence in CHAT systems. This ordinal rating system is intended to evaluate the quality of evidence for responses to guiding questions in Table 1

Measurement				Assessment Design			
Qualitative data		Quantitative data		Alignment		Sampling	
QL.0	Data have not been collected.	QT.0	Data have not been collected.	A.0	Data and question are clearly unaligned, or no data have been collected	S.0	Data have not been collected. Information is solely based on researchers’ perceptions.
QL.1	Data have been collected. However, construct validity has not been formally evaluated or analyses were done incorrectly.	QT.1	Data have been collected. However, construct validity has not been formally evaluated or analyses were done incorrectly.	A.1	Data have face validity. Criterion validity has not been formally evaluated or analyses were done incorrectly.	S.1	Data were collected through convenience sampling. Helps generate hypotheses but inferences cannot be made beyond sample.
QL.2	Formal evaluations of construct validity have been conducted. However, evidence for both internal or external validity is limited, or studies were not conducted in your context of interest	QT.2	Formal evaluations of construct validity have been conducted. However, evidence for both internal or external validity is limited, or studies were not conducted in your context of interest	A.2	Formal evaluations have been conducted and provide some evidence for criterion validity.	S.2	Data were collected intentionally. However, sampling may have occurred at an incorrect scale, there is sampling bias, or participants choose not to participate.
QL.3	Formal evaluations of construct validity have been conducted. There is strong evidence for both internal or external validity in your context of interest	QT.3	Formal evaluations of construct validity have been conducted. There is strong evidence for both internal or external validity in your context of interest	A.3	Formal evaluations have been conducted and provide strong evidence for criterion validity.	S.3	Data were collected using a random sample of the population (or sample frame) of interest or data exists for the entire population.

**Table 3:** Example responses to the CHAT questionnaire (Table 1) for assessing participant selection into the Harvard Forest Summer Research Program in Ecology

Question (Table 1)	Response	Relative priority to evaluate	Quality of evidence (Table 2)
S.0a	HF‐SRPE is a paid summer research program for undergraduate participants located at the Harvard Forest in Petersham, Massachusetts, USA. Over the past 30 years, HF‐SRP has been primarily supported through the NSF REU program; however, additional participants within the program have been supported through various grants and programs. There are numerous stakeholders in the hiring process who, under a more extensive evaluation, would receive their own activity system: applicants, referees for applicants, researcher mentors, and program administrators	High	QL.1 QT.0 A.1 S.1
S.0b	Same as above. Since this is an example of internal evaluation, we have a working relationship with all program staff. Information about applicants	Low	QL.1 QT.0 A.1 S.0
S.1	Yes. However, since this is an internal evaluation, it will be important to record the relevant context so that others can adequately understand the nuances of the activity system	High	QL.1 QT.1 A.1 S.1
S.3	There have been subtle changes in program structure and funding over the past 30 years. We have an archive of general descriptions of the program through grant documents and advertising material; however, a formal document analysis has not been conducted to address this question	Medium	QL.1 QT.0 A.1 S.1
S.4	An activity system can/should be created for each stakeholder in this process. Individual activity systems naturally group around the collective action of hiring for an individual position, followed by projects supported by multiple REU positions, and finally, HF‐SRP. However, the applicants and mentors may also perceive aspects of this activity system differently based on their experiences or knowledge of similar *activity systems*: Other REU programs and field ecology research experiences; hiring for research positions; undergraduate and graduate admissions	Medium	QL.0 QT.0 A.0 S.0
Ob.0a	Select participants for the Harvard Forest Summer Program in Ecology	High	NA
Ob.0b	Equitable hiring process. Support interests such as providing research opportunities to undergraduate students who can benefit the most from the program, supporting research programs of PIs, the mission of the Harvard Forest	High	QL.1 QT.0 A.1 S.1
Ob.0c	Mentors may have similar perceptions of the selection process. However, they could possibly prioritize aspects such as speed or ease of hiring process, demonstrated skills of applicant (as opposed to potential). This has not been examined directly and would warrant further investigation	High	QL.1 QT.0 A.1 S.1
Ob.1	Overall, there seems to be alignment between act of hiring for various positions. However, since there are many stakeholders there may be conflicting values and expectations based on the individual positions (rules)	High	QL.1 QT.0 A.1 S.1
Ob.2	Individuals within the *community* may have different priorities shaped by their own values and expectations for the position (see below).	Low	QL.0 QT.0 A.0 S.0
Ob.3	The need to select participants for the program has remained the same (although values, rules, mediation artifacts, etc. have changed; see below)	Low	QL.0 QT.0 A.0 S.0
Ob.4	Undergraduate admissions, graduate admissions, other REU programs: Members of the *community* may perceive aspects of this activity system differently based on their experiences/knowledge of similar *activity systems*	Low–medium	QL.0 QT.0 A.0 S.0
Out.0a	HF‐SRPE wishes to support equitable hiring practices, promote diversity in the field ecology, and select participants who can benefit the most from the program	High	QL.1 QT.0 A.1 S.1
Out.0b	Primarily only demographic information (gender, ethnicity, institution type, first‐generation status)	High	QL.1 QT.1 A.1 S.3
Out.1	We have not identified any contradictions between the measurement and our values associated with the hiring process	Medium	QL.0 QT.0 A.0 S.0
Out.2	The demographic information is broadly aligned with most of the goals of the program. However, with regard to inclusive hiring practices, these data only provide a little bit of insight into an applicant's personal identity	High	QL.0 QT.0 A.0 S.0
Out.3	Due to the simplicity of these items, most of these measurements have not changed	Low	QL.0 QT.0 A.0 S.0
Out.4	The data we collect are very similar to that of adjacent *activity systems*	Low	QL.0 QT.0 A.0 S.0
C.0	Undergraduate applicants, principle investigators, HF‐SRPE staff, HR	Low–medium	QL.4 QT.1 A.1 S.4
C.1	Mostly, however, HF‐SRPE has a difficult time identifying participants who do not apply to the program (but might otherwise turn out to be competitive applicants). Without knowing who those individuals are, or their barriers to application, HF‐SRPE is limited in its ability to determine if it is providing an equitable application process	Medium–high	QL.0 QT.0 A.0 S.
C.2	Applicants will know their referees (and anyone else helping them with their application) and the PIs on listed projects. However, they are likely unaware of all the members of the research team and program staff who are involved in the hiring process Program staff and research mentors are most likely to work together during this process. Research mentors have the ability to review to all student applications, although typically review those who indicate a preference for their project	Low–medium	QL.0 QT.0 A.0 S.0
C.3	On the administrative side, it is often different each year with some overlap in administrative staff and mentors. Sometimes, there are repeat applicants and referees	Medium	QL.0 QT.1 A.1 S.3
C.4	Based on the nature of some projects, there may be a larger community involved during the hiring process (e.g., more applicants, larger research groups) Applicants may also receive support from their home institutions (faculty mentors, advisors, peers) constructing application materials We also have anecdotal evidence that our *community* is similar in structure to that of other REU programs. However, we do expect that the size of our applicant pool (~800 undergraduate annually), PIs, and program staff are greater than most REU sites	Medium‐High	QL.1 QT.1 A.1 S.1
R.0	Rules and expectations of this hiring process are akin to most other employment opportunities. Although mentors can set their own expectations for hiring criteria, HF‐SRP program staff try to unify program expectations (e.g., projects involving intellectual participation of the participant, sharing equitable hiring resources, communicating expectations of funding agencies). HF‐SRP also provides logistical advice for applicants to clarify program expectations for both the application process and the summer experience	High	QL.0 QT.0 A.0 S.0
R.1	We have tried to be consistent in advertising material, hiring expectations, and values. However, we have not conducted formal evaluations to see whether the messages we send are aligned with our values (e.g., explicitly review all advertising material and requirements through a multicultural lens)	High	QL.0 QT.0 A.0 S.0
R.2a	There do not appear to be any rules or admission requirements that would limit us from hiring participants for all available positions	Low–medium	QL.0 QT.0 A.0 S.0
R.2b	We have not observed any rules that would prevent the community from functioning. *Community* members within the activity system may have conflicts with the rules, expectations, and cultural norms of the hiring process. However, these are better addressed by examining that individual's activity system (which this would then be a secondary contradiction between the subject, rules, and community)	Low	QL.0 QT.0 A.0 S.0
R.3	Rules, deadlines, and expectations are routinely changing as we reflect more on the hiring process. For example, over the past 15–20 years, we have increased our commitment to recruiting a diverse applicant pool. These values have helped shape improvements to mediating artifacts such as recruitment methods and the hiring process	Medium–high	QL.0 QT.0 A.0 S.0
R.4	For the most part, rules are applied consistently, and we do not expect to see contradictions between activity systems of different subjects We believe that HF‐SRPE’s rules and expectations are comparable to other REU programs or internship programs. However, it is possible that there are cultural norms establish by other programs that are in contrast with HF‐SRPE and may be a barrier to recruiting certain qualified participants	Med	QL.0 QT.0 A.0 S.0
MA.0	Advertising materials (position advertisements, program website) Submission tool (front‐end used by applicants and references to submit application materials) Submission tool (back‐end used by mentors and HF‐SRPE staff to review applicants) Hiring documents (project descriptions, interview protocols, expectations)	High	QL.1 QT.0 A.1 S.2
MA.1	These *mediating artifacts* have undergone an iterative design process over many years; however, a formal analysis has not been conducted to see whether the *mediating artifacts* are aligned with each other	High	QL.1 QT.0 A.1 S.1
MA.2a	These *mediating artifacts* have undergone an iterative design process over many years; however, a formal analysis has not been conducted to see whether the *mediating artifacts* are aligned with the *object*	Medium	QL.0 QT.0 A.0 S.0
MA.2b	The perceived utility of *mediating artifacts* will vary based on the subject and their role in the process. We have not received any notable complaints about *mediating artifacts* but do not formally collect this type of feedback	Medium	QL.0 QT.0 A.0 S.0
MA.2c	In general, we believe that members of the *community* are finding the *mediating artifacts* useful. Over the years, we have received informal feedback which has guided the design of our submission tools and hiring procedures. However, we are still uncertain how our recruiting process/materials might be limiting otherwise qualified applicants from applying	Medium	QL.1 QT.0 A.1 S.1
MA.2d	We have not received feedback indicating that our *rules* (or *division of labor*) conflict with any of the *mediating artifacts*	High	QL.0 QT.0 A.0 S.0
MA.3	We believe that the introduction of certain *mediating artifacts* (e.g., online submission tool) have been beneficial to improving *outcomes* such as diversity in hiring	Medium–high	QL.0 QT.0 A.0 S.0
MA.4	Due to the high volume of applications (>500 per summer) and the digital infrastructure already in place at the Harvard Forest, HF‐SRPE has invested a fair amount of effort into our online submission tool. We imagine that smaller (or newer) programs do not have the need for such infrastructure. While we feel that these mediating artifacts are important for HF‐SRPE to achieve our goals, we anticipate that there are many successful alternatives	Medium–high	QL.1 QT.0 A.1 S.1
DL.0	Application materials: HF‐SRPE staff create and disperse most of these materials; mentors may also disperse materials through their own networks; some applicants may need to seek out these materials, while others may be introduced to them through their network of peers, faculty, or career services programs Submission tool—front‐end: Applicants submit their own materials, but they may be aided in this process by peers, faculty, or career services programs. Applicants also indicate their top interests in projects Submission tool—back‐end: HF‐SRPE program director reviews all applicants prioritized by established funding sources. This provides a second set of eyes on hundreds of applications and reduces some of the burden on mentors hiring for individual projects Hiring documents: Program mentors have some autonomy in how they describe project descriptions, set expectations for desired experience, and conducting interviews. HF‐SRP program staff provide resources and accountability to help ensure that mentor decisions are aligned with the program's goals and values	High	QL.1 QT.0 A.1 S.1
DL.1	Expectations of *division of labor* are seemingly understood by most *community* members but not formally examined.	Medium	QL.1 QT.0 A.1 S.1
DL.2a	We routinely receive feedback from the *community* and believe that there is an adequate *division of labor* to support the *mediating artifacts*	Medium	QL.1 QT.0 A.1 S.1
DL.2b	We routinely receive feedback from the *community* and believe that there is an adequate *division of labor* to support the *object*	Medium	QL.1 QT.0 A.1 S.1
DL.3	The largest change to the *division of labor* occurred when the program director started doing a preliminary review of applicants. This aided mentors in the early stages of the hiring process and helped relieve some of the burden of reviewing an increasing number of applicants	Medium–high	QL.1 QT.0 A.1 S.1
DL.4	HF‐SRPE likely has similar *division of labor* compared to other REU programs, especially regarding application materials and hiring documents. However, due to the extraordinarily high number of applications HF‐SRPE receives a year, the achievement of *outcomes* such as diversity in hiring may be more sensitive to changes in the *division of labor*	Medium	QL.0 QT.0 A.0 S.0

**Table 4:** Example responses to a CHAT questionnaire (Table 1) for assessing participant learning gains from the Harvard Forest Summer Research Program in Ecology

Question (Table 1)	Response	Relative priority to evaluate	Quality of evidence (Table 2)
S.0a	The *subjects* are individual participants in the HF‐SRPE program who come to Harvard Forest from a range of undergraduate institutions. Since research mentors hire participants for specific research projects (e.g., plant ecology, soil microbiology, biogeochemistry, paleoecology, programming or data science), participants bring with them a variety of educational experiences. Additionally, some participants may be specifically selected based on their skillsets (or lack thereof) based on structure of project goals during the 11‐week program. For example, some projects may be structured in a way that allows participant to learn and explore with limited scientific skills or knowledge, whereas other projects may require participants to have a specific set of skills or background knowledge to generate a specific research product within the 11‐week time period	High	QL.1 QT.1 A.1 S.4
S.0b	*Subjects* would generally characterize themselves in a similar manner. However, with regard to scientific skill or knowledge, they may not be able to self‐evaluate their abilities (especially on novel skills or concepts)	Low–medium	QL.0 QT.0 A.0 S.0
S.1	There is somewhat of a consensus about the *subjects*; however, data characterizing these *subjects* is highly dependent on self‐report surveys	High	QL.1 QT.1 A.1 S.3
S.3	We have two types of self‐report data that help characterize the *subjects*: The HF pre‐/postsurvey (begun ~ 15 years ago) and some questions on URSSA (begun ~ 8 years ago). Additionally, application materials are archived going back ~ 15 years but we would need additional IRB approval to access information beyond simple demographics	Medium	QL.1 QT.1 A.1 S.3
S.4	There are multiple types of academic, research, and professional experiences that are tangential to HF‐SRPE and may influence how participants perceive or approach this program: other REU experiences, independent research at home institution, laboratories associated with coursework, STEM courses	Medium	QL.0 QT.0 A.0 S.0
Ob.0a	There are many types of learning gains that HF‐SRPE is interested in, however, for the purpose of this example we will choose to focus only on evaluating how HF‐SRPE helps participants improve their quantitative reasoning (specifically focusing on collecting, visualizing, analyzing, and communicating ecological “Big Data”)	High	NA
Ob.0b	Some participants may come from programs where this is not emphasized, have an aversion to math, or simply may not understand the important role of quantitative reasoning in research. They may not be able to characterize certain aspects of quantitative reasoning prior to the program (i.e., novice perspective)	High	QL.0 QT.0 A.0 S.0
Ob.0c	Other than peers, most members of the HF‐SRPE *community* (mentors, researchers, staff) recognize the importance of quantitative reasoning in research and would consider it a priority to learn during HF‐SRPE	Low	QL.1 QT.0 A.1 S.1
Ob.1	Generally, there is a clear consensus about this being an important goal for participants in the program	Low	QL.1 QT.0 A.1 S.1
Ob.2	Improving quantitative reasoning may not be a priority for all participants. This may be more common on the few projects that do not involve classical ecological research such artists in residence and social science projects	High	QL.0 QT.0 A.0 S.0
Ob.3	In general, promoting quantitative reasoning has been a consistent across all years; however, emphasis on collecting, visualizing, analyzing, and communicating ecological “Big Data” is the theme of the most recent REU Site award. Before this was a central theme for the entire program, this was an emphasis of some student projects so there were staff and resources available prior to broader deployment	Low	QL.0 QT.0 A.0 S.0
Ob.4	We imagine this is a common goal in many REU programs as well as STEM courses, laboratories, and other research/internship experiences. However, from our experience from HF‐SRPE, we know that the emphasis of this goal by students and research mentors can vary widely based on the nature of a student's research project	Low	QL.0 QT.0 A.0 S.0
Out.0a	HF‐SRPE is hoping that participants develop quantitative reasoning skills that persist with participants well after this program is completed. We seek to promote broad practices that can be applied anywhere from a scientific research career to an informed citizen	High	NA
Out.0b	Currently, quantitative reasoning is broadly assessed through an internally developed self‐report survey and through a few items in URSSA	High	QL.1 QT.1 A.1 S.3
Out.1	The self‐report survey has not been validated. URSSA has been validated for broad understanding of quantitative skills at the program level and admit these questions are not robust at the individual level	High	QL.1 QT.1 A.1 S.3
Out.2	We do not have strong evidence of participants' ability to collect, visualize, analyze, or communicate data	High	QL.0 QT.0 A.0 S.0
Out.3	We have had multiple versions of internally developed self‐report surveys over the years. URSSA has been consistent for the past ~8 years	Medium	QL.1 QT.1 A.1 S.3
Out.4	Current instruments were designed for use on REU programs or HF‐SRPE. However, to improve our assessment, we will need to rely on instruments developed outside of the REU context	Medium	QL.0 QT.0 A.0 S.0
C.0	Participants will interact with mentors, peers, research team members, and seminar or workshop presenters; however, this will vary based on the project on which the participant is working	Low	QL.1 QT.0 A.1 S.1
C.1	Yes, due to the nature of the working relationship, the *subject* will be quite familiar with the *community*	Low	QL.0 QT.0 A.0 S.0
C.2	For the most part, both the *subject* and *community* will know each other. There may be some instances where some seminar/workshop presenters may not know some/most of the *subjects*	Low	QL.0 QT.0 A.0 S.0
C.3	Each year, the *community* is different. Some mentors and seminar or workshop presenters may remain, but new *subjects* are selected each year. We can only really track *subject's* growth over an 11‐week period	Low	QL.0 QT.0 A.0 S.0
C.4	Although the division of labor may change, most students share this same community. We also imagine this *community* structure may be similar to other coordinated research programs but will likely differ considerably compared to independent research and coursework	Medium	QL.0 QT.0 A.0 S.0
R.0	There are many cultural norms and conventions associated with collecting, visualizing, analyzing, or communicating data and these norms may also change within subdisciplines. We teach participants R and there is a certain amount of fluency necessary to interact with this coding language	Medium–high	QL.0 QT.0 A.0 S.0
R.1	We have not examined whether *rules* conflict with one another. We would imagine rules associated with specific mediating artifacts are internally consistent but can easily conflict with each other	Medium–high	QL.0 QT.0 A.0 S.0
R.2a	Although we have not formally evaluated this, the *rules* seem to generally promote learning quantitative reasoning	Low	QL.0 QT.0 A.0 S.0
R.2b	Yes, this is quite possible. Depending on their training, some members of the community may not possess the same quantitative reasoning background (e.g., we teach the R programming language, but the mentor may not know it)	Medium	QL.0 QT.0 A.0 S.0
R.3	HF‐SRP receives feedback from members of the *community* and routinely adjusts rules, expectations, and social norms based on this feedback. We do not have a consistent record of these changes	Medium	QL.0 QT.0 A.0 S.0
R.4	Depending on a student's project, there might be different values and expectations with regard to “Big Data.” It is quite possible that adjacent *activity systems* (e.g., computer science degree program vs. ecology degree program) have different conventions. Depending on the concepts and the participant's strength of adoption, it may be difficult for them to accommodate new conventions	Medium‐High	QL.0 QT.0 A.0 S.0
MA.0	R workshops Scientific communication workshops Project proposals Project specific research activities (different for each individual)	High	QL.1 QT.1 A.1 S.1
MA.1	*Mediating artifacts* are developed independently by various member of the community. While they may understand program goals and the needs of certain projects, they have not been intentionally aligned during design	Medium–high	QL.0 QT.0 A.0 S.0
MA.2a	In general, we believe the *mediating artifacts* align with the *object*. However, we have limited information on *mediating artifacts* developed solely by mentors and do not know how they would align	Medium–high	QL.0 QT.0 A.0 S.0
MA.2b	The *subjects* view of *mediating artifacts* varies. For example, we know that participants hired to work on highly computational projects do not gain much from the introductory R workshops	High	QL.0 QT.0 A.0 S.0
MA.2c	It is somewhat unclear how the *community* perceives the utility of the *mediating artifacts*	Low–medium	QL.0 QT.0 A.0 S.0
MA.2d	It is possible that some *rules* associated with individual *mediating artifacts* in conflict with other *mediating artifacts*. As for *division of labor*, we know that some *subjects* engage with *mediating artifacts* differently and may take on new roles (e.g., experienced coders may act as peer tutors). When this occurs, the *division of labor* and expectations of the *subject* change	Medium	QL.0 QT.0 A.0 S.0
MA.3	*Mediating artifacts* are routinely introduced and updated. We have not yet examined how this impacts *outcomes,* but we anticipate most changes to make the overall program run smoother and hopefully improve *outcomes*	Medium	QL.0 QT.0 A.0 S.0
MA.4	For many participants, their previous coursework or research experiences had not prepared them in this manner. Even for those who have had more exposure to quantitative methods, most have not had such a holistic curricula. Because of this variability, students may interact with the same mediating artifact in vastly different ways	Medium	QL.0 QT.0 A.0 S.0
DL.0	Our *mediating artifacts* are designed to take some of the burden of teaching these quantitative skills off the mentors, many of whom may not have the time or background to do so themselves. Within a *mediating artifact*, there may be different expectations based on a *subject's* previous experience. For example, some *Subjects* may act as peer facilitators while others may need to spend much more of their spare time becoming fluent in a programming language (as there are often steep learning curves)	High	QL.0 QT.0 A.0 S.0
DL.1	We are often unaware if *community* members are not meeting their expected *division of labor.* The two exceptions would be workshop facilitators (as participants complete multiple evaluations) and participant who does not show up or are clearly not engaged. Many *subjects* and *community* members are within these two extremes and shortcomings could easily fall under the radar	Medium	QL.0 QT.0 A.0 S.0
DL.2a	We can often glean from seminar or workshop evaluations if the *division of labor* is not appropriate. Once again, it is much easier to detect the extremes and we rely on the facilitators to find the correct balance	Medium	QL.1 QT.0 A.1 S.1
DL.2b	Since our *outcome* measures are not ideal for measuring many aspects of quantitative reasoning, it is unclear how appropriate the *division of labor* is across all the *mediating artifacts*	High	QL.0 QT.0 A.0 S.0
DL.3	As we have introduced and developed *mediating artifacts*, we know the *division of labor* has changed but we have a limited record of such change	Low–medium	QL.0 QT.0 A.0 S.0
DL.4	Given the time constraints of an 11‐week program and the expectations on participants, we anticipate that the *division of labor* is different compared to adjacent *activity systems*. Based on project needs and student backgrounds, facilitators may spend more time helping participants learn basic concepts at the beginning of the program, but student may require much more autonomy at later stages of the program when they are working on project specific activities	Low–medium	QL.0 QT.0 A.0 S.0

**Table 5:** Example responses to the CHAT questionnaire (Table 1) for assessing long‐term program impacts of the Harvard Forest Summer Research Program in Ecology

Question (Table 1)	Response	Relative priority to evaluate	Quality of evidence (Table 2)
S.0a	We tend to attract undergraduate students primarily interested in environmental or ecological careers (as opposed to health professions). Many of our undergraduate students are unclear about their specific career objectives (especially if they are first‐ or second‐year students). For some, this is their first formal research experience and it helps them explore what they like (or dislike) about scientific research. For others with more research experience or firmer career objectives, they take the opportunity to learn skills that will help prepare them for graduate school or the workforce	High	QL.1 QT.0 A.0 S.1
S.0b	We imagine students would characterize themselves like we have above; however, we have not asked them to do so for assessment purposes	High	QL.0 QT.0 A.0 S.0
S.1	We are confident in correctly characterizing basic descriptive information (e.g., major, institution type, demographics); however, we anticipate characterizing career intentions to be difficult. At this point in their undergraduate career, students have many paths to choose from. As they learn about and explore various career options, we imagine that their intents may fluctuate	High	QL.0 QT.0 A.0 S.0
S.3	We anticipate that over time, there may be shifts in demands for various skillsets (e.g., working with “Big Data”). As a program, we try to respond to these trends by adjusting projects and programming. It is reasonable to think these changes may lead to a change in how students are characterized	Medium	QL.1 QT.0 A.0 S.1
S.4	There are multiple types of academic, research, and professional experiences that are tangential to HF‐SRPE and may influence how they perceive or approach the program: other REU experiences, independent research at home institution, laboratories associated with coursework, STEM courses	Medium	QL.0 QT.0 A.0 S.0
Ob.0a	Since 2010, Congress has mandated that we monitor the long‐term impact of REU programs. For congress, these goals appear to be focused on building a strong STEM workforce. We generally believe that persistence in STEM is an important; however, we also recognize that careers in education/outreach, policy, or simply being a more scientifically literate citizen are equally valid long‐term goals	High	NA
Ob.0b	It is somewhat unclear what individual students might state as their long‐term goals, but for most, we anticipate that their goals are related to graduate school/employment. However, they may have an alternate perception of success	High	QL.1 QT.0 A.0 S.1
Ob.0c	We anticipate that research mentors have similar views of long‐term success for students. However, as researchers, they may have additional goals such as establishing long‐term collaborations with students (either as future graduate students or colleagues)	Medium	QL.1 QT.0 A.0 S.1
Ob.1	While it will depend on the individual student, it is quite possible that there is not a consensus on the long‐term goals. It is likely that most of these disagreements will be between the HF‐SRPE, student, or mentor and congressional goals, causing secondary contradictions between the *object* and *outcomes* mandated by the America COMPETES Act of 2010	High	QL.0 QT.0 A.0 S.0
Ob.2	Since students apply to take part in HF‐SRPE, we would expect that most of their long‐term goals are in alignment with the program. However, tensions could arise during *mediating artifacts* if the intent was not aligned with the *subject's* perception of long‐term goals (i.e., a student with no interest in graduate were required to write or participate in a workshop about graduate school admission essays)	Medium	QL.0 QT.0 A.0 S.0
Ob.3	We do not anticipate large cultural shifts in long‐term goals	Low	QL.0 QT.0 A.0 S.0
Ob.4	Similar goals are common throughout U.S. culture; however, students may be a part of a community or home institution that may hold a different set of values	Low	QL.0 QT.0 A.0 S.0
Out.0a	Success may mean multiple things to multiple people. Just because long‐term success is not achieved under one metric, does not mean that a student did not have a successful experience. Additionally, there are many other factors that contribute to the “long‐term success” of students and it would be presumptuous to think that an 11‐week program is the only factor leading to this metric. Additionally, people have different paths to success and may appear more or less successful depending on when the measurements are taken	Medium	NA
Out.0b	Our assessment priority for long‐term success will be dictated by the America COMPETES Act of 2010 which requires the tracking of students for STEM matriculation and employment for at least three years following graduation. We have an annual survey that is sent to all program alumnae(i) (since 2001) that tracks enrollment and attainment of degrees, and STEM employment	High	NA
Out.1	This *outcome* is a requirement of our funding agency and HF‐SRPE has the discretion to choose how we measure the *outcome*. Our current measurement relies on self‐report data. We have a convenience sample which decreases over time as we lose track of former students (e.g., out of date contact information, name changes)	High	QL.1 QT.1 A.1 S.1
Out.2	Our *outcome* measures only have face validity	Medium	QL.0 QT.0 A.1 S.0
Out.3	Our alumni survey has remained relatively consistent since 2010	Low	QL.1 QT.1 A.1 S.0
Out.4	We developed this survey in‐house but have been exploring other techniques to track these *outcomes*. One promising avenue for improvement is to transition to techniques used by college alumni associations	Medium	QL.0 QT.0 A.0 S.0
C.0	Students will interact with mentors, peers, research team members, and seminar or workshop presenters; however, this will vary based on the project on which the student is working	Low–medium	QL.1 QT.0 A.1 S.1
C.1	Yes, because of the nature of the working relationship, the *subject* should be quite familiar with the *community*	Low	QL.0 QT.0 A.0 S.0
C.2	For the most part, both the *subject* and *community* will know each other. There may be some instances where some seminar or workshop presenters may not know some or most of the *subjects*	Low	QL.0 QT.0 A.0 S.0
C.3	Each year the *community* is different. Some mentors and seminar or workshop presenters may remain, but new *subjects* are selected each year. We can only really track each *subject's* growth over an 11‐week period	Low	QL.1 QT.0 A.1 S.1
C.4	We imagine this *community* structure may be like that of other coordinated research programs but will likely differ considerably compared to independent research and coursework	Medium	QL.0 QT.0 A.0 S.0
R.0	Our goal is to treat students as employees and colleagues rather than “undergraduate students.” The aim is to model professional behavior and provide support to students (for some of whom, this is their first job) so that they eventually feel a sense of autonomy and accountability for their actions	Medium–high	QL.1 QT.0 A.1 S.1
R.1	Since students are living at the Harvard Forest, there is sometimes difficulty delineating work from recreation. Social norms are established between housemates, peers, and supervisors that are unique to each cohort. This is challenging to navigate, and it is very easy for individuals to receive social signals that are at odds with the intent of the program	Medium–high	QL.1 QT.0 A.1 S.1
R.2a	Although we try to establish consistent rules and expectations, there are instances where rules and norms may contradict each other. This often happens in response to an incident at work or in the residences that require enforcement of rules and policies. In these instances, the actions of a few individuals may cause the group to feel a loss of autonomy	Low	QL1 QT.0 A.1 S.1
R.2b	No, students bring with them their own set of values which could be at odds with the norms that HF‐SRPE is trying to establish. We try to mitigate these conflicts by being transparent about the rationale for certain expectations and open to dialogue (although this is easier said than done).	Medium	QL.0 QT.0 A.0 S.0
R.3	Although the intent is to be consistent with rules, expectations, or cultural norms from year to year (modeled after the Harvard Forest research community), there is inherently some variability	Medium	QL.1 QT.0 A.1 S.1
R.4	HF‐SRPE has a similar feel to academic research cultures on traditional campuses; however, there are many aspects related to a rural biological field station that create a distinct set of rules and expectations. For example, feelings of isolation and irritability (i.e., cabin fever) are common among students and we try to be cognizant about how we provide support to students as they transition to the new environment (and the norms associated with it)	Medium–high	QL.0 QT.0 A.0 S.0
MA.0	We provide two structured and informal opportunities for students with regards to graduate school and career opportunities Career panel Networking with researchers Other structured activities focus on broader skills useful for scientific careers Independent research project (with a research mentor) Project proposals Science communication workshop Blogging Poster workshop R programming workshop Research seminars Additionally, there are many more “one‐off” opportunities that are created by research mentors or based on student interest. These are often in response to an individual's summer/career goals	High	QL.1 QT.0 A.1 S.3
MA.1	When designing the programming, we consider how professional development activities support students with respect to the mission of the program and student long‐term goals	Medium‐High	QL.1 QT.1 A.1 S.2
MA.2a	We continuously solicit feedback from students and mentors to monitor how students’ short‐term and long‐term goals are being supported	Medium–high	QL.1 QT.1 A.1 S.2
MA.2b	Students perception of utility often depends on their skillsets or experiences. When designing an activity, we try to accommodate a range of skill levels	High	QL.1 QT.0 A.1 S.1
MA.2c	The *community* generally finds these professional development activities useful. However, individual research mentors may prioritize research above some required activities	Low–medium	QL.0 QT.0 A.0 S.0
MA.2d	In general, we believe that our *rules* align these *mediating artifacts* and how they are accomplished	Medium	QL.0 QT.0 A.0 S.0
MA.3	Based on feedback, we continuously introduce, remove, or revise *mediating artifacts*. It is unclear how these changes are related to long‐term success	Medium	QL.0 QT.0 A.0 S.0
MA.4	These *mediating artifacts* are common to other research programs and educational settings. We find that even if a student has completed a similar activity before, the repetition is useful as they may gain a different perspective the second (third or more) time around	Low	QL.0 QT.0 A.0 S.0
DL.0	The research mentor and student are asked to outline expectations for the project proposal at the beginning of the summer. *Division of labor* is somewhat variable among research mentors which is why HF‐SRPE provides formal programming to help ensure a consistent exposure to professional development resources. Sometimes, a student will maintain a research relationship with their mentor after the end of the summer (often resulting in a research project such as a poster, undergraduate thesis, or manuscript) For other professional development activities, HF‐SRPE strives to provide resources for students that their research mentors may not otherwise have the time or expertise to provide	Medium	QL.1 QT.0 A.1 S.1
DL.1	Although we have a proposal that outlines expectation for each student's project, we do not revisit these documents to evaluate whether the agreed upon *division of labor* was met. Our reluctance to analyze these documents is due to how these documents are formatted (some projects require a lot of structure while others are more trial‐and‐error) and that research goals may change rapidly throughout the summer	High	QL.0 QT.0 A.0 S.0
DL.2a	Most of the time, students and mentors find the *division of labor* is appropriate. However, we do have mentors and students come to program staff when they feel that expectations are not being met. Program staff act as mediators to resolve any conflicts and provide alternative to help both side move forward in a productive manner	Medium	QL.0 QT.0 A.0 S.0
DL.2b	Currently it appears that the *division of labor* throughout the program is appropriate	Medium–high	QL.1 QT.0 A.1 S.1
DL.3	Project proposals were introduced in response to student feedback that the *division of labor* was not being meet by some research mentors. The introduction of this *mediating artifact* helped to clarify expectations	Low–medium	QL.1 QT.0 A.1 S.1
DL.4	For both students and research mentors, the *division of labor* may be different from what they are used to in other settings. We stress that HF‐SRPE students have intellectual involvement in the project and should not be viewed as grunt labor. This may cause a shift in how some individuals view their role and we try to provide support to aid in such a transition	Medium–high	QL.0 QT.0 A.0 S.0

### 4.1 Program context

The Harvard Forest Summer Research Program in Ecology (HF‐SRPE) (https://harvardforest.fas.harvard.edu/other-tags/reu), provides paid, mentored research experiences in field‐ or laboratory‐based ecology, plant biology, and forestry to 20–30 students each year. In recent years, students also have linked research in software engineering and robotics, and communication and outreach to broader ecological topics and concepts. Through three decades, we have participated in the development and implementation of NSF’s vision for REU sites. Simultaneously, we have enhanced student experiences and improved short‐ and long‐term effectiveness of our program by regularly measuring and reflecting on its success and failures and integrating our assessment data with REU‐wide evaluations (McDevitt et al., 2016). We also have recognized that theoretical frameworks such as CHAT improve our formative and summative program evaluation. Below, we provide three examples that are valued by our program and that we continually devote significant time toward improving: equitable recruitment and hiring practices, participant learning gains, and persistence in STEM.

### 4.2 Recruitment and hiring practices

**Figure 3:**
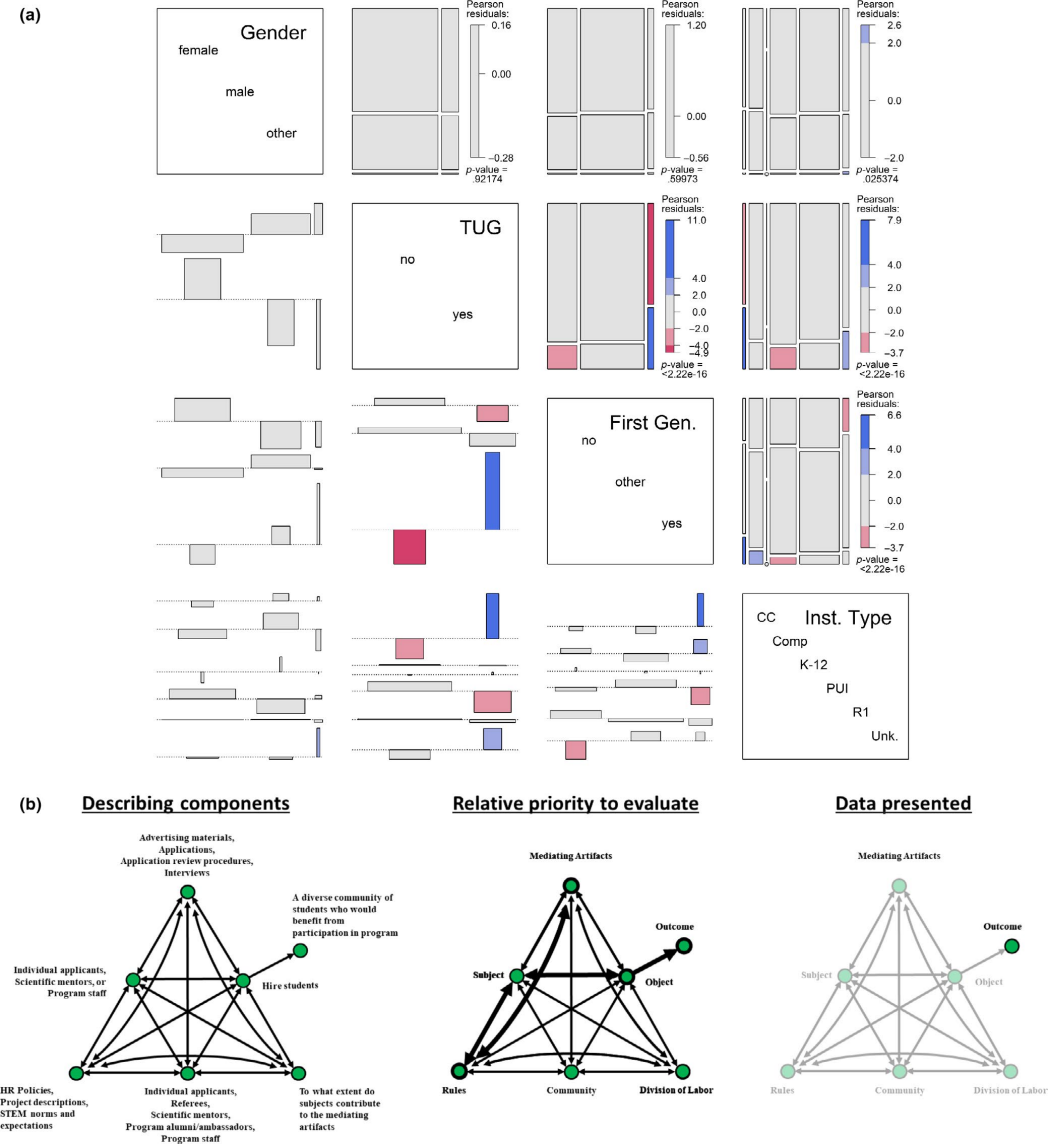
Data from applicants (top) to the Harvard Forest Summer Research Program in Ecology (HF‐SRPE). Data like these are commonly collected during the recruitment and hiring process by all undergraduate research programs. (a) Pairwise visualization of conditional independence models for four demographic variables: Gender (female, male, other [including undeclared and nonbinary]); TUG: Student from groups traditionally under‐represented in science; First.Gen: Students who are the first in their family to attend college or university; Inst.Type: type of institution, including community college (CC), comprehensive university (Comp), K‐12 (kindergarten through high school), PUI (primarily undergraduate institution), R1 (research‐1 university), and Unk (unknown or not applicable). The demographic variables and their possible values are shown along the diagonal. The panels above the diagonals are *mosaic plots* (Hartigan & Kleiner, 1984) that illustrate the observed frequencies of the *y* variable conditional on the *x* variable. For example, the plot of Gender (*y*) versus TUG (*x*) illustrates the frequencies of female, male, or other‐gendered individuals conditional on whether each individual is from a group traditionally underrepresented in science. The area of each tile is proportional to the corresponding cell entry given any previous conditioning. Continuing with the Gender versus. TUG example, we first conditioned on TUG (the *x* variable); there have been more non‐TUGs than TUGs in the Harvard Forest Summer Research Program in Ecology, so the width of the “no” group is much larger than that of the “yes” group. We then split Gender conditional on TUG; there are many more females than males, and few nonbinary individuals. The shading (red to grey to blue) is proportional to the residual from a χ^2^ contingency table (*i.e.,* difference of observed from expected values); the overall *P* value for the χ^2^ test is given below the vertical residual scale‐bar. In the Gender versus TUG example, the residuals are small, and there is no significant relationship in our hiring of students of different genders given their ethnicity (*p* = .92). The panels below the diagonals are *association plots* (Cohen, 1980). As with the mosaic plots, the association plots illustrate differences from expectation of the *y* variable conditioned on the *x* variable. Rather than illustrating the observed frequencies, the association plot illustrates the standardized deviations of observed frequencies from the expected frequencies. The direction of each rectangle from the dotted (zero) line indicates the sign of the residual; its height is proportional to the magnitude of the residual; its width is proportional to the square root of the expected counts; and its area is proportional to the difference between the observed and expected frequencies. Colors match those of the mosaic plots. Plot constructed with the pairs() function within the vcd library in R (Meyer, Zeileis, & Hornik, 2006). (b) CHAT activity triangles (Figure 2) that show how components could be assessed with current frameworks (bottom right) or within a full CHAT framework (bottom center and bottom left)

The first stage of all REU programs, including the HF‐SRPE, is the recruitment and hiring of a diverse cohort of student participants (Figure 3a). Recruitment and hiring for HF‐SRPE is a collective action that requires submission of materials by participants and referees, and hiring decisions by program staff, scientists, and administrators (*subjects*). For simplicity, we have constructed an *activity triangle* (Figure 3b) representing the entire recruitment and hiring process, as opposed to constructing activity triangles for each individual's contributions to hiring a single participant. We describe characteristics about the HF‐SRPE (*subject*), the process of hiring participants for these positions (*object*), the expectations of the hiring process (*rules*), and practices implemented in recruiting or selection (*mediating artifacts*) to recruit and hire a diverse set of students with various degrees of prior experience participating in mentored research (*outcome*).

At these earliest stages of the program, *primary contradictions* exist in establishing the priorities for recruitment (*object*). We try to strike a balance between selecting students who appear to be best qualified (i.e.*,* most experienced) to do research and those who have the most to gain out of the experience. These contradictions arise in part from cultural biases of academic research where success is measured through productivity (theses, posters, peer‐reviewed papers); the “best” students are those with proven “track records” of productivity. As mentors and educators, we also want to work with students who are willing to push beyond their comfort zone and maximize the impact of a research experience. At HF‐SRPE, this *primary contradiction* is further complicated by the different stakeholders involved in the hiring process. Individual research mentors advocate for their projects; funders push for students from certain institutions, demographics, academic majors, or skillsets; and program directors seek a lasting and cohesive identity for the program.

At HF‐SRPE, we have sought to balance the *quaternary contradictions* between activity systems of multiple stakeholders (including the program directors, program manager, mentors, external collaborators, and funders) by building research teams (*mediating artifact*). Research teams consist of multiple mentors and multiple students who work together to address scientific inquiries through complementary collaborations. Stakeholders meet to discuss the formation of research teams prior to creating a position (*mediating artifact)* and project roles are adjusted to create peer leadership opportunities based on diverse skillsets; graduate students may take on additional mentorship roles (often acting as a “near‐peer” mentors). These activities establish clear hiring expectations (*rules*) for applicants and mentors and increase equity during the process of reviewing applications (*object)*. Once applications are submitted, program directors review and filter the applicant pool (*mediating artifact)* to increase alignment among project needs, broader program goals, and hiring requirements stipulated by funders (*rules*). Mentors selecting students from this filtered subset of applicants meet programmatic and project requirements (*rules*) by hiring students with skillsets and the potential to gain additional value from the experience. This two‐step applicant review process, although time consuming, provides additional oversight that helps guard against implicit biases that might cause us to overlook applicants who can contribute to research outcomes *and* benefit from the research experience.

Another barrier to recruiting and hiring a diverse population of students are *secondary contradictions* between potential applicants (*subjects*), application materials (*mediating artifacts*), and the norms surrounding finding an internship (*rules*)*.* Reviewing recruitment and application materials through a multicultural lens is a continual process and has been our primary tool for limiting these *secondary contradictions*. However, an unanticipated recruitment strategy of the HF‐SRPE has been to take advantage of positive research experiences our students have. They tell others about the experience at their home institutions, conferences, and meetings, through social media outlets, and forward emails/promotional material (*mediating artifacts*)*.* In some cases, they have returned to HF‐SRPE as mentors.

Characterizing these various components and assessing whether recruitment and hiring goals are being met is especially difficult when nearly 1,000 applications are reviewed in less than four weeks. Applying CHAT to HF‐SRPE’s requirement and hiring practices (Table 3) has helped organize and prioritize our thoughts. Hiring for REU positions in an equitable way requires minimalizing contradictions across the activity systems of multiple stakeholders. Although it would be best to collect lines of evidence supporting each CHAT component, an evaluation of our priorities (Table 3) has highlighted the need to consider how applicants from diverse backgrounds may perceive and interpret recruitment and application materials (*secondary contradictions* between s*ubjects, mediating artifacts,* and *rules*); the priorities of the mentors filling each position (s*econdary contradictions* between *subjects, rules,* and *object*); the expectations of the site PIs in recruiting a diverse group of participants (*rules*); and the final hiring decision (*outcome*).

Currently, the most consistent information we and most other REU sites collect about the hiring process are demographic data and quantifiable metrics such as gender, ethnicity, grade‐point average (GPA), class rank, and type of institution (Figure 3a). These data are relatively easy to gather from applicants, can influence decisions about who to interview or hire, and are straightforward to track through time or compare among multiple REU sites. The data illustrate that our applicants are predominantly white, female, and from a mix of institutional backgrounds (Figure 3a). Conditional models suggest little difference from expectation except that our applicants who are the first in their family to attend college tend to be from ethnic groups broadly underrepresented in science and attend either community colleges or comprehensive universities (Figure 3a). However, these data align with only a few of the priorities highlighted by CHAT and are insufficient for accurate evaluation and assessment. While we have carefully thought about and tried to address these priorities, this reflective exercise reveals that we should integrate additional information into our formative and summative program evaluations; evaluate the effectiveness of recruitment materials by analyzing their messages through a multicultural perspective (Dumas‐Hines, Cochran, & Williams, 2001; Pippert, Essenburg, & Matchett, 2013); consider how our application requirements and selection criteria may be biased against student populations we wish to serve (Ployhart & Holtz, 2008); and review how tools, procedures, and policies impact the division of labor among various stakeholders (students, mentors, program administrators, program leadership).

### 4.3 Understanding variation in learning gains

**Figure 4:**
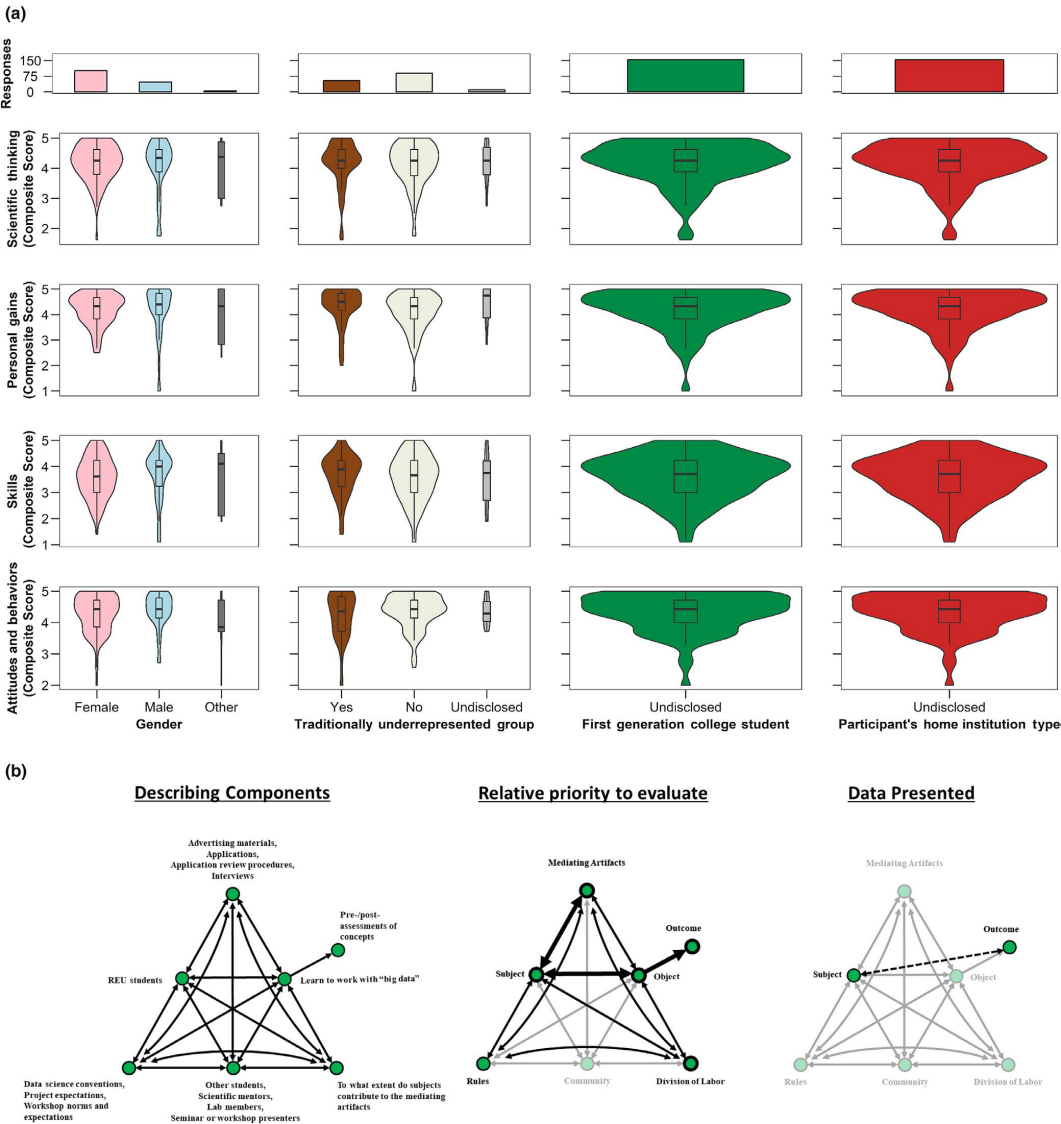
Data commonly collected when assessing learning gains (top) and CHAT activity triangles illustrating how components could be assessed with current frameworks (bottom right) or within a full CHAT framework (bottom center and bottom left). The top panels show changes in scientific thinking, personal gains in overall confidence in doing research, research skills, and attitudes and behaviors about doing research among students participating in the Harvard Forest Summer Research Program in Ecology (HF‐SRPE). Values range from 1 (low) to 5 (high) for all variables. The total number of participants in the different groups is shown in the top row; in the other panels, violin plots show the distribution of the data with inset box plots illustrating median, quartile, and upper and lower deciles of the data. Additional analysis of these data aggregated with additional data collected from pre‐/postsurveys given annually to undergraduate participants in REU sites supported by the Biological Sciences directorate (BIO) at NSF are presented in Weston and Laursen (2015)

The scientific theme for the most recent five‐year (2015–2019) REU Site award for the HF‐SRPE was the collection, visualization, analysis, and communication of ecological “Big Data.” Like other REU sites in biology, we have used URSSA (Hunter et al., 2009) to provide self‐assessment of gains in learning through questions about broad items related to thinking and working like a scientist. URSSA includes questions that address students’ attitudes, feelings, and motivation related to analyzing data for patterns, problem‐solving, and identifying limitations. Superficially, these may seem like they can assess the learning gains of interest, but the developers of URSSA defined its scope only as a broad indicator of progress (Weston & Laursen, 2015). The questions are not aligned with our specific program goals (i.e., they have “poor criterion validity”) and are unable to provide meaningful measurements for any of our “Big Data” learning outcomes. Additionally, the limited student or programmatic context provided by URSSA, such as demographics, rarely accounts for much of the variation in URSSA’s measured gains (Figure 4). Such limitations have constrained our ability to improve the HF‐SRPE or assess whether we are helping students achieve defined goals.

We describe components and contradictions within a CHAT activity system (Table 4) to identify more useful data to address the learning objectives of the HF‐SRPE. Although it would be ideal to align and characterize all seven components of the activity system with respect to learning gains, we set priorities for assessment characterizing the skills and knowledge a student brings with them to the research experience (*subject*); the resources used by the student during their research experience (*mediating artifacts* such as R workshops or project proposals); the level of support they received (*division of labor*); and what success (*object*) means given a student's prior research experience. These priorities align with the idea that the tools individuals use to construct knowledge are culturally mediated (Vygotsky, 1980; Wertsch, 1993). For example, although two students may participate in the same R workshop (*mediating artifact)*, their prior experiences and the workshop's relevance to their project may fundamentally shape how they interact with the activity (*secondary contradictions* between the *subject*, *rules*, and *mediating artifact*). The variation in student projects also means that students may be interacting with different resources or using them to different extents (*quaternary contradictions* in *mediating artifacts* and *division of labor*). We recognize that the program's “Big Data” learning goals may not be a priority for all students (i.e., *secondary contradictions* between the *subject object,* and *outcome*) and “success” may mean something completely different to them than what it does to their mentors or the overall HF‐SRPE. Even without characterizing all components and interactions, this richer characterization of the learning environment provides greater insights into learning gains (*outcome*).

We have limited evidence to support how HF‐SRPE facilitated learning gains related to “Big Data” because of differences in the assessment data collected in the past (McDevitt et al., 2016) and priorities identified by CHAT. This disconnection results partly from an under‐described activity system for each student and partly from a lack of sufficient measures for this learning goal (i.e., URSSA; Hunter et al., 2009). Searches for a concept inventory (i.e., a validated educational instrument for evaluating student ideas and beliefs about a topic) that aligned with our program's “Big Data” learning objectives (*object*) have been unsuccessful, partially because data science concept inventories (e.g., Allen, 2006; Caceffo, Wolfman, Booth, & Azevedo, 2016) are typically designed to assess concepts specific to statistics and computer science coursework. Since developing a new concept inventory is difficult to justify without broader applicability (e.g., coordination across programs with similar objectives), we are left with the following options: continue using student self‐evaluations while acknowledging that students are likely to have difficulty evaluating a topic in which they are not yet proficient; depend on mentor evaluations of student proficiencies that also depend on the mentors’ proficiency and their familiarity with students’ progress; or do a detailed analysis of research products while recognizing that these may not represent the breadth of what a student learned.

### 4.4 Assessing the impact of REU programs on persistence in STEM

**Figure 5:**
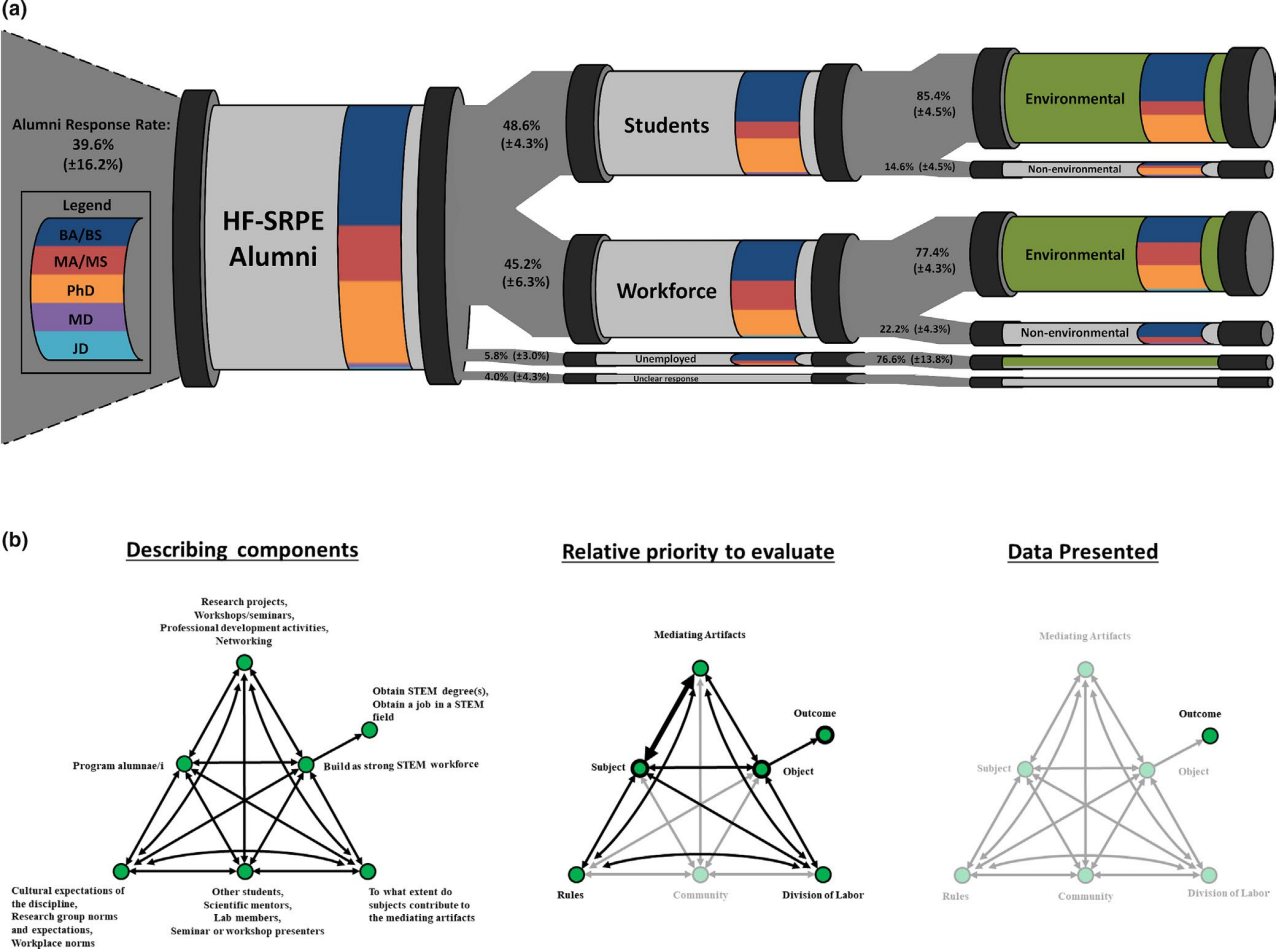
Career outcomes (“pipeline”) of participants in the Harvard Forest Summer Research Program in Ecology (HF‐SRPE). Annual alumni surveys were sent to alumnae(i) (cohorts from 2001 onward) between 2012 and 2016. Averages of yearly snapshots reveal that most alumnae(i) have pursued or received environmental‐ or ecology‐related graduate degrees and continue to use these disciplines during their careers. Further information is required to determine the impact of HF‐SRPE on these outcomes. The CHAT activity triangles (bottom) illustrate how components could be assessed with current frameworks (bottom right) or within a full CHAT framework (bottom center, bottom left)

Feedback from previous HF‐SRPE participants have suggested that mentored independent research is a formative experience for their career development. Systematic, postprogram tracking of REU participants remains challenging despite it being a legal requirement in the US since 2010 (P.L. 111‐478 §514). We annually survey past participants of HF‐SRPE; the resulting data provide some support for long‐term persistence and high rates of employment by HF‐SRPE alumnae(i) in STEM fields (Figure 5). However, we have been unable to account accurately for the distribution of nonresponses or determine specific effects of HF‐SRPE on individual decisions to pursue STEM careers. Like many of our colleagues who work with REU students, we believe that mentored research experiences launch them into STEM careers, but we cannot predict where they would be without this experience. Ethical and logistical constraints prevent researchers from forming true control groups for REU participants; we can use only quasi‐experimental designs. Again, we turn to guided data collection efforts before and during REU programs that can help to meaningfully characterize students and their experiences.

It is currently difficult for us to disentangle the impact of the HF‐SRPE and selection bias. Although our recruitment practices aim to recruit students who would benefit the most from this experience, we cannot discount that our participants have successfully demonstrated their potential for research in a highly competitive application process. Additionally, participants have other formative experiences that impact the long‐term persistence in STEM disciplines or careers. To help generate hypotheses about how HF‐SRPE may impact a participant's persistence in STEM, we used the CHAT framework (Table 5) to prioritize the collection of the following data: the skills and knowledge a student brings with them to the research program (*subject*); the professional development opportunities available to them during their research experience (*mediating artifacts*); the interactions students have with other members of the research community (*community*); and the goals of the research experience (*object*).

As with our applicants, much of the information we have for program alumnae(i) is related to basic demographic information. Additionally, for alumnae(i), we have information on research products and responses to annual surveys. Reporting program impacts to our funders has focused primary on the annual surveys, but these occur after students have participated in the HF‐SRPE and collect only data on educational level or attainment and employment status. We therefore know little about why our students do or do not persist in STEM disciplines or careers.

Other research provides evidence that students participating in structured undergraduate research programs obtain advanced degrees and generate research products at a higher rate than a matched cohorts of students, but an understanding about how or why this occurs is limited (Wilson et al., 2018). Given the priorities identified by CHAT, we would want to collect data that help explore hypotheses related to the procedures and cultural expectations (*rules*) that determine who is selected to participate in HF‐SRPE or other REU sites (biasing for characteristics that may be independent of basic demographic descriptors such as gender, ethnicity, home institution, or GPA); specific *mediating artifacts* that help students achieve their career goals (recognizing that there are likely multiple equivalent paths to long‐term success); and acknowledging how students (*subjects*) and other members of their *community* may view and support success (*outcome*). Collecting rich data to explore these mechanisms would likely require ethnographic interviews (e.g., Carlone & Johnson, 2007; Hernandez & Morales, 1999). Although this type of study would certainly prove useful as formative program evaluation, the amount of time and resources needed would not make it practical to collect at the same scale of our annual surveys.

## 5 CONCLUSIONS

We have provided three examples that demonstrate the flexibility of CHAT for framing the study and assessment of different aspects of REU programs: recruitment and hiring practices, student learning gains, and the impact on participant persistence in STEM. CHAT provided an opportunity to reflect upon the complex educational system that is an REU site in a way that allowed us to connect with existing sociocultural frameworks. Examining HF‐SRPE’s hiring practices required us to consider the activity systems of all individuals contributing to the process and the *quaternary contradictions* between similar activity systems. This was a slightly different approach from when examining learning gains related to “Big Data.” There, the emphasis was directed more to the students (*subject*) and the application of CHAT focused on *secondary contradictions* that might hinder students from achieving the learning goal. Finally, when applying CHAT toward the impact HF‐SRPE may have on participant's persistence in STEM, we considered the different opportunities students may have had during their REU experience (*quaternary contradiction*) and acknowledge that we have limited information about the activity systems of other experiences that might also shape a participant's persistence in STEM.

Based on our positive experiences, we advocate for integration of sociocultural frameworks such as CHAT in assessment and evaluation. This systems approach has proven useful for studying other complex educational phenomena by helping derive meaning from seemingly contradictory information (Daniels, Edwards, Engeström, Gallagher, & Ludvigsen, 2013; van Oers, Wardekker, Elbers, Veer, & eds., 2008; Talbot et al., 2016). As with most scientific inquiry, the research questions ultimately should drive the types of data that are collected. However, we believe that CHAT is broad enough that it can guide the summative and formative evaluations for most aspects of REU programs. Meaningfully engaging with this framework requires both a clear understanding of programmatic goals and a familiarity with the theory and literature in education research. However, we have found that spending time characterizing activity systems has helped us to formalize our thinking and evaluate the alignment of our programmatic priorities with our assessment tools.

Characterizing components of any activity system and examining its contradictions can help identify barriers to success within it (Engeström, 1987, 2001). REUs are complex activity systems, and characterizing and connecting them to established theoretical frameworks should make it easier to transfer novel ideas and best practices across the larger REU community. Applying these principles to the HF‐SRPE has revealed to us that we are overemphasizing our data collection efforts on *subject‐object‐outcome* while ignoring *artifacts, communities, division of labor*, and *rules*. This is limiting because the REU experience is a sociocultural experience that takes place within nested or articulating communities and those communities are socially, culturally, and historically influenced. As evaluative research continues to develop within the REU community, we see systems‐based theoretical frameworks as useful guidelines for programs to follow when assessing REU programs.

REU programs provide an opportunity for students to work and learn with experienced researcher(s) and develop a community with their peers. The social and cultural experiences of REUs are its greatest strength, but REUs can potentially fail students when the social–cultural–historical underpinnings of the program are not given their due. REU students must navigate sociocultural contexts, which in turn should influence how REU sites are designed and implemented. Sociocultural frameworks such as CHAT provide a systems‐based perspective that helps characterize and identify important components and interactions within the complex learning environment.

## CONFLICT OF INTEREST

Aaron Ellison and Manisha Patel are the founding principals of Sound Solutions for Sustainable Science LLC, which provides project management for scientific education and research programs. We do not advocate for any particular theoretical framework in managing, evaluating, or assessing research and education programs.

## AUTHOR CONTRIBUTIONS

Andrew McDevitt collected data; analyzed data; and contributed to writing the manuscript. Manisha Patel is an HF‐SRPE program manager; collected data; and contributed to writing the manuscript.

Aaron Ellison is an HF‐SRPE PI/PD; obtained funding; developed conceptual framing; analyzed data; and contributed to writing the manuscript.

## Data Availability

Data reported in this paper are available from the Harvard Forest Data Archive (http://harvardforest.fas.harvard.edu/data-archive/), datasets HF279, 280, and 281, with respective dois of: https://dx.doi.org/10.6073/pasta/f74630916b73ce5d5c023bdf8fd5692c; https://dx.doi.org/10.6073/pasta/641d056f549b8c2fc0507165087697ff; and https://dx.doi.org/10.6073/pasta/10b8e12edf5a203d0598fd0d9d3ffda6. Protocols for collecting data from student participants in the HF‐SRP were approved by the Committee on the Use of Human Subjects (Institutional Review Board) at Harvard University (IRB file numbers IRB14‐2580 and IRB15‐2719). Informed consent was obtained from all student respondents.
